# Image-guided sonoporation in an *ex vivo* machine perfused porcine liver

**DOI:** 10.1186/2050-5736-3-S1-P99

**Published:** 2015-06-30

**Authors:** Christina Keravnou, Christophoros Mannaris, Maria-Louisa Izamis, Michalakis Averkiou

**Affiliations:** 1 University of Cyprus, Nicosia, Cyprus

## Background/introduction

Sonoporation is the transient and reversible cell membrane permeability change induced with ultrasound and microbubbles. It allows for the uptake of normally impermeable macromolecules and has been suggested for improving drug delivery. The exact sonoporation mechanism and the optimal ultrasound parameters are still under investigation. *Ex vivo* machine perfused porcine livers are an excellent platform for investigating the sonoporation parameters and specifically the interaction of ultrasound driven microbubbles with the capillaries. Our objective was to identify the ultrasound parameters that are capable of causing detectable perfusion changes in the sonoporation area. Three types of perfusion changes were considered: large mechanical damage void of perfusion, reduced perfusion due to capillary destruction, and unaltered perfusion.

## Methods

**Figure 1:**
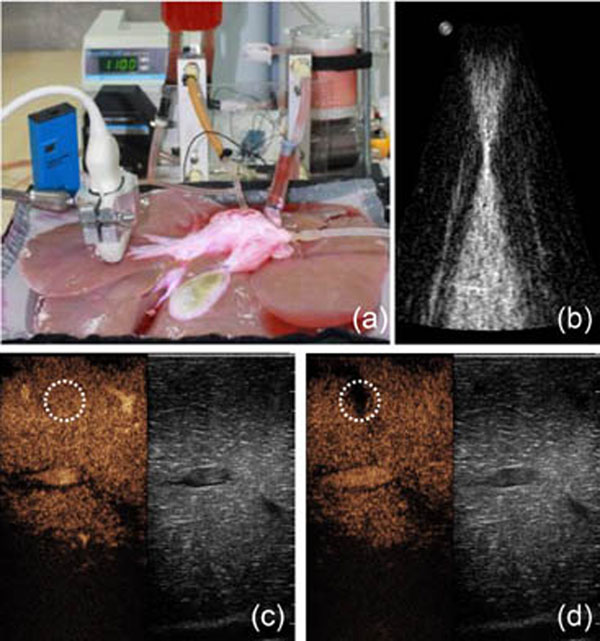
(a) *Ex vivo* pig liver machine perfusion system; (b) Therapy transducer RF interference signal picked by the imaging probe; (c) Perfusion image before sonoporation; (d) Perfusion image after sonoporation showing a perfusion defect.

Porcine livers were collected from a local slaughterhouse and connected to a machine perfusion system [Fig. 1(a)]. Injections of experimental contrast microbubbles (BR38, Bracco Suisse SA) in either the portal vein or the hepatic artery were followed with ultrasound treatment of a specific area. Ultrasound pulses at 1 MHz and varying durations, pressures, and duty cycle (<10% to avoid heating), were fired by single element transducers (focused and unfocused). The process was monitored with diagnostic ultrasound (C5-1 probe, Philips iU22). The therapy transducer spatial extend was accurately overlaid in the ultrasound images by a novel technique where the RF noise of the power amplifier was transmitted through the therapy transducer and detected by the diagnostic ultrasound probe [Fig. 1(b)]. The perfusion of the treated area was evaluated with Dynamic Contrast Enhanced Ultrasound (DCEUS) quantification methods.

## Results and conclusions

Perfusion change caused by image-guided sonoporation [Fig. 1(c) baseline, (d) after sonoporation] was demonstrated in an *ex vivo* machine perfused liver with a combined therapy-imaging system. The use of unfocused therapy transducers led to a much larger treatment area and was easier to identify and measure perfusion changes with DCEUS. Focused therapy transducers produced higher acoustic pressures but at smaller areas that were difficult to identify with DCEUS unless the focused transducer was mechanically scanned to treat a larger area. Sonoporated areas with ultrasound pressure above 1 MPa showed a detectable perfusion change. Complete mechanical damage was present at much larger acoustic pressures (~10 MPa). Shorter acoustic pulses (50 cycles) produced less perfusion changes than longer pulses (500 cycles) for the same duty cycle.

